# Differential somatostatin sensitivity of corticotropic and somatotropic responses following amino acid infusion

**DOI:** 10.1530/EC-26-0537

**Published:** 2026-09-24

**Authors:** Elvar Már Sturlaugsson Jóhönnuson, Josephine Reiche, Michael M Richter, Nicolai J Wewer Albrechtsen

**Affiliations:** ^1^ Department of Clinical Biochemistry, Copenhagen University Hospital – Bispebjerg and Frederiksberg, Copenhagen, Denmark; ^2^ Copenhagen Center for Translational Research, Copenhagen University Hospital – Bispebjerg and Frederiksberg, Copenhagen, Denmark; ^3^ Department of Clinical Medicine, Faculty of Health and Medical Sciences, University of Copenhagen, Copenhagen, Denmark

**Keywords:** hormone dynamics, hypothalamus, hypothalamic–pituitary–adrenal axis, neuroendocrine function, somatotropic axis

## Abstract

**Objective:**

Amino acids stimulate pituitary hormone secretion, but whether somatostatin-sensitive pathways differentially regulate activation of the corticotropic and somatotropic axes during amino acid infusion remains unclear. We investigated whether somatostatin signalling constrains growth hormone secretion following amino acid infusion differently from ACTH and cortisol responses in humans.

**Methods:**

We conducted a randomized, within-subject crossover study in 15 healthy adults with three morning visits: amino acid infusion plus saline, somatostatin analogue infusion plus saline, and combined amino acid plus somatostatin analogue infusion. Vamin, an amino acid mixture, was infused at 331 mg·kg^−1^·h^−1^ from 0 to 45 min, and octreotide, a somatostatin analogue, was infused at 200 ng·kg^−1^·min^−1^ from −75 to 165 min; blood was sampled from −75 to 300 min. *Post hoc* exploratory outcomes included baseline-subtracted area-under-the-curve measures for cortisol, ACTH, and growth hormone, analysed using repeated-measures mixed-effects models.

**Results:**

Following amino acid infusion, ACTH and cortisol showed rapid responses, peaking around 45 min, whereas growth hormone increased gradually and peaked around 120 min. Compared with somatostatin analogue alone, amino acid infusion increased peak concentrations and baseline-subtracted exposures for cortisol, ACTH, and growth hormone. Co-infusion with somatostatin analogue substantially reduced ACTH and cortisol responses but almost completely abolished growth hormone secretion.

**Conclusion:**

Corticotropic and somatotropic responses were observed following intravenous amino acid infusion in humans, with markedly different somatostatin sensitivity. Growth hormone secretion was almost completely suppressed by somatostatin analogue administration, whereas ACTH and cortisol responses remained partly preserved, suggesting that HPA axis activation can partly escape somatostatin inhibition.

**Significance statement:**

Amino acids are commonly viewed as nutritional substrates, but they can also act as endocrine stimuli. In this study, intravenous amino acid infusion was followed by activation of both the corticotropic and somatotropic axes in healthy adults, producing rapid ACTH–cortisol responses and a delayed growth hormone response. The findings show that these responses differ markedly in their sensitivity to somatostatin signalling: growth hormone secretion was almost completely suppressed by somatostatin analogue administration, whereas ACTH and cortisol responses remained partly preserved. These findings provide physiological insights into how corticotropic and somatotropic responses following amino acid infusion differ in their sensitivity to octreotide.

## Introduction

Adrenocorticotropic hormone (ACTH) and growth hormone (GH) are secreted from anterior pituitary cells under hypothalamic control through corticotropin-releasing hormone (CRH), arginine vasopressin (AVP), and growth hormone-releasing hormone (GHRH). ACTH regulates adrenal cortisol secretion and the physiological stress response (1), whereas growth hormone has central metabolic and anabolic functions (2, 3). Somatostatin, produced predominantly in the hypothalamus, acts as a major inhibitory regulator of pituitary secretion, particularly of GH release, primarily through somatostatin receptor subtype 2 (SSTR2)-mediated suppression of GHRH signalling (4). In contrast, the effects of somatostatin on ACTH secretion appear less pronounced and more variable.

Several amino acids and amino acid mixtures stimulate pituitary hormone secretion. Arginine is well established as a GH secretagogue (5, 6) and also stimulates vasopressin/copeptin secretion (7), whereas its effects on ACTH and cortisol appear context dependent (8). An oral amino acid mixture has been reported to enhance ACTH responsiveness to CRH/GnRH and to be associated with a higher total GH AUC (9), while excitatory amino acid signalling has been implicated in the regulation of ACTH release (10). Another amino acid mixture has been shown to suppress the exercise-induced cortisol response (11).

Amino acid solutions, such as Vamin, are widely used in parenteral nutrition, while somatostatin analogues, such as octreotide, are frequently administered in individuals with gastrointestinal and neuroendocrine disorders. Understanding how amino acid-driven endocrine responses interact with somatostatin signalling is relevant to pituitary physiology and nutrient-induced endocrine regulation.

The present study is a post hoc exploratory analysis of a randomized crossover trial previously reported with respect to pancreatic islet hormone and metabolic responses (12). Here, we examined ACTH, cortisol, and growth hormone concentrations measured during the same experimental visits to compare corticotropic and somatotropic response patterns following amino acid infusion and their sensitivity to octreotide.

## Methods

### Study design and approvals

Data for the present analyses were derived from a previously published randomized within-subject crossover trial investigating amino acid-induced pancreatic islet hormone and metabolic responses (12). ACTH, cortisol, and growth hormone were measured during the same experimental visits but were not prespecified outcomes in the original study protocol. No prospective statistical analysis plan or sample size calculation was developed for these outcomes. The statistical analyses described below were developed specifically for the present *post hoc* exploratory analysis and were not part of the parent study protocol.

The study was open-label, and participants and investigators were not blinded to treatment allocation. Each participant attended three visits at least five days apart. Two intravenous catheters were placed, one in each antecubital vein: one for infusion and one for repeated blood sampling. The participants completed three experimental days in randomized order (Fig. 1): i) octreotide infusion (*t* = −75 to *t* = 165 min) + saline (*t* = 0 to *t* = 45 min) (due to one participant’s absence, only 14 individuals completed the octreotide + saline study day); ii) Vamin infusion (*t* = 0 to *t* = 45 min) + saline (*t* = −75 to *t* = 165 min); and iii) octreotide + Vamin (co-infusion) according to the above time frames.

**Figure 1:**
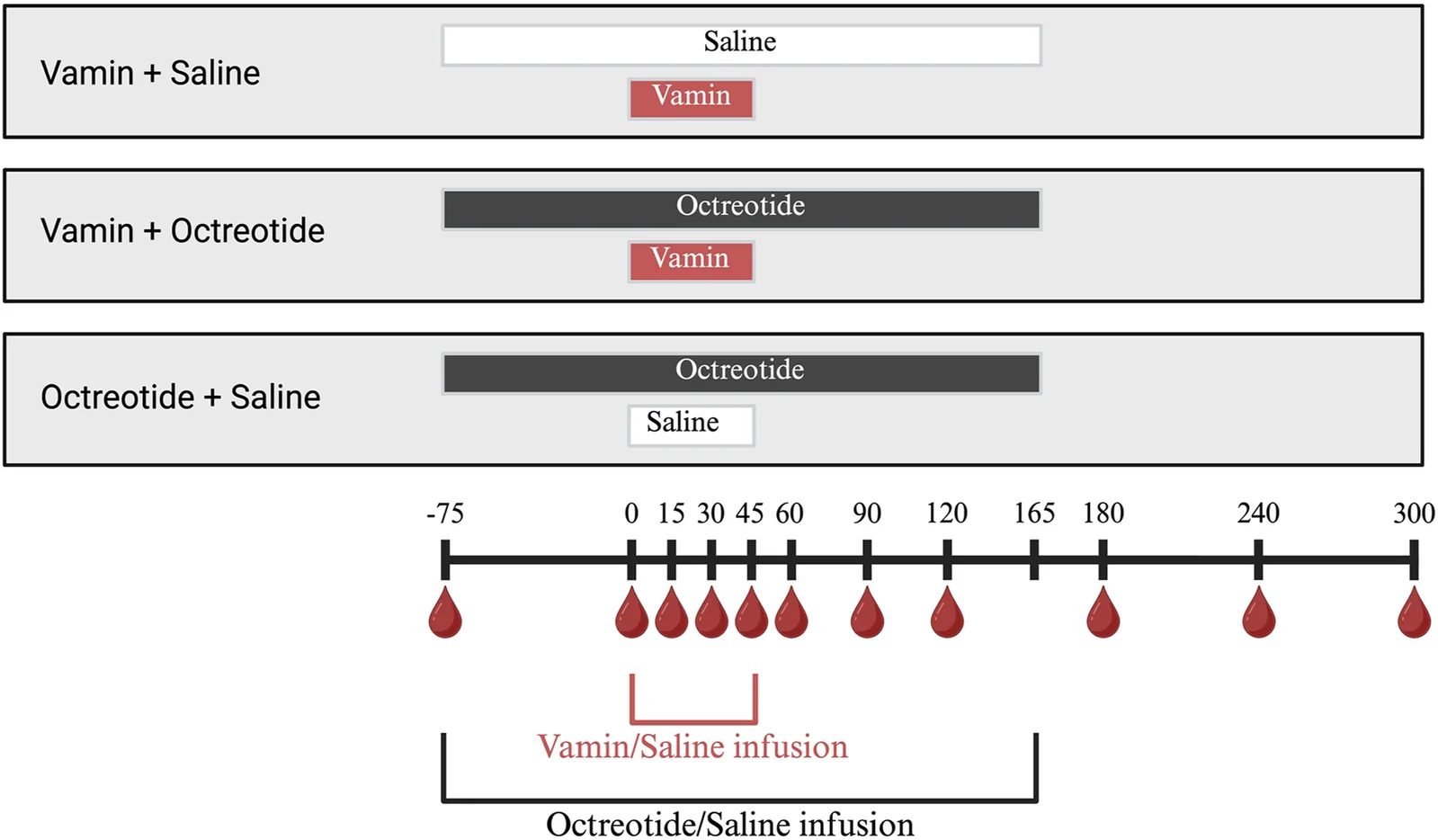
Study schedule and sampling. Participants completed three morning visits in a randomized order: octreotide + saline infusion day, Vamin + octreotide infusion day, and Vamin + saline infusion day. Octreotide infusions started at −75 min and ended at 165 min; meanwhile, Vamin was infused from 0 to 45 min. Venous blood was sampled at variable time points from −75 to 300 min. Analyses include *n* = 15 participants.

This study was conducted in 2024 and 2025 at the Department of Clinical Biochemistry, Bispebjerg Hospital, Copenhagen, Denmark. It was approved by the Scientific-Ethical Committee of the Capital Region of Denmark (registration number H-23036057) and the Danish Data Protection Agency (Privacy, registration number p-2023-24424). The trial was registered at ClinicalTrials.gov (NCT06240039). The study was conducted in accordance with the principles of the Helsinki Declaration, and informed consent was obtained before inclusion.

### Participants

Eligible participants were lean individuals aged 25–65 years with a body mass index (BMI) ≤ 25 kg/m^2^. Screening included medical history; measurement of height, weight, and body composition; and laboratory tests (including haemoglobin, HbA1c, ALT/AST, and creatinine/eGFR). Biocomposition was assessed by bioelectrical impedance (Tanita MC-780MAP).

Exclusion criteria were diabetes mellitus (type I or II) or HbA1c above the reference range; pregnancy or breastfeeding; current smoking; known cardiovascular disease; active or recent malignant disease; history of alcohol or substance abuse; impaired liver or kidney function; current medication use other than contraception; acute illness within the preceding two weeks; or any other disorder that, in the opinion of the investigator, was incompatible with participation. No participant reported glucocorticoid use. Shift-work status was not systematically assessed and was not an exclusion criterion.

Pre-visit restrictions were enforced to minimize physiological variability. Participants fasted for 10 h before each study day and abstained from alcohol and strenuous exercise for 48 h. Water was permitted until arrival. Nonadherence led to rescheduling. Participants were encouraged to maintain their usual routines during the days leading up to the study days to maximize comparability between the three visits. Recruitment was done via advertisements on the Trialtree platform.

### Procedures

The participants arrived at 08:00 am after a 10-h overnight fast and abstinence from alcohol and strenuous exercise for 48 h. Infusions started at approximately 09:00 am (±15 min) to reduce variability from circadian HPA axis activity. Participants remained in the clinical research unit throughout the study and did not consume food or beverages.

At time −75 min, participants received a continuous intravenous infusion of octreotide (Pfizer, New York, USA) or saline for 240 min. Octreotide was initiated 75 min before Vamin administration to establish its inhibitory endocrine effect before the amino acid challenge. Octreotide was infused at 200 ng·kg^−1^·min^−1^, capped at 1,000 μg/h. At time 0 min, participants received either Vamin Electrolyte-Free, a 14 g N/L amino acid solution (Fresenius Kabi, Denmark), infused at 331 mg·kg^−1^·h^−1^ for 45 min, or saline for 45 min. Blood samples were collected at −75, 0, 15, 30, 45, 60, 90, 120, 180, 240, and 300 min.

At each time point, two tubes were collected: one 4-mL VACUETTE® Lithium Heparin tube (Greiner Bio-One, Germany) sent directly to the laboratory for analysis (<30 min) and one 4-mL VACUETTE® K2-EDTA tube (Greiner Bio-One, Germany), which was centrifuged, after which plasma was isolated, and stored at −20°C and −80°C until analysis.

### Assays

Plasma ACTH, cortisol, and GH were analysed using sandwich electrochemiluminescence immunoassays on a cobas 8000 (Roche Diagnostics, Switzerland). Cortisol was measured at all 11 sampling time points (−75, 0, 15, 30, 45, 60, 90, 120, 180, 240, and 300 min), whereas ACTH and GH were measured at −75, 0, 45, 120, and 300 min. Measurements of plasma glucose, glucagon, insulin, c-peptide, and total amino acids have been described previously (12).

### Statistical analysis

For each participant and visit, the concentration at time 0 was subtracted from all subsequent concentrations. Time 0 was selected because it marked the start of the Vamin or saline intervention following 75 min of octreotide or saline pretreatment. Signed baseline-subtracted areas under the curve were calculated using the linear trapezoidal method, with values below baseline retained as negative contributions. The analysis windows were defined as 0–45 min for cortisol, 0–120 min for ACTH, and 0–300 min for GH. These windows were selected to capture the principal response periods observed for each hormone and were not prespecified in the original study protocol.

Across all study visits, 10 ACTH, 16 cortisol, and 10 GH values were missing. One participant did not complete the octreotide + saline visit, accounting for 5 missing ACTH values, 11 missing cortisol values, and 5 missing GH values, consistent with the hormone-specific measurement schedules described above. The remaining five values for each hormone were missing at isolated time points during otherwise completed visits. Peak and bsAUC outcomes were analysed separately using repeated-measures mixed-effects models fitted by restricted maximum likelihood in GraphPad Prism, version 10.4.1. Condition was included as a fixed effect, and participant was included as a random effect. Although treatment order was randomized, period and treatment sequence were not included in the model, and no formal tests of period or sequence effects were performed. The model therefore estimates within-participant differences between conditions without adjustment for potential period or sequence effects. Prism’s implementation uses a compound-symmetry covariance structure. Sphericity was not assumed, and the Geisser–Greenhouse correction was applied. Pairwise comparisons were adjusted using Tukey’s method. Pairwise differences were reported as the first-listed condition minus the second-listed condition such that positive values indicated a higher response in the first-listed condition. No separate sample size calculation was performed for the present exploratory outcomes. Data distribution and homoscedasticity were assessed by visual inspection of Q–Q plots and residual plots.

Eight ACTH and 29 growth hormone measurements were below the assay lower limit of quantification (LLOQ) and were set to the LLOQ (0.7 pmol/L and 0.05 μg/L, respectively) before analysis. Tables and figures display mean ± SEM. Outliers identified by the ROUT method (*Q* = 0.1%) were removed.

## Results

### Participants

We enrolled 15 healthy adults (8 men and 7 women) aged 25–35 years (mean: 27 ± 2.7 years). The mean BMI was 21.6 ± 2.1 kg/m^2^. Baseline glycaemic and liver fibrosis indices were normal (fasting plasma glucose: 5.6 ± 0.4 mmol/L, HbA1c: 34 ± 2.4 mmol/mol, FIB-4 score: 0.67 ± 0.26) (Table 1). No adverse events occurred. Baseline characteristics have previously been described (12).

**Table 1:** Baseline characteristics of study participants. Data are presented as mean ± SD.

Characteristics	Values
Demographics	
Total *n*	15
Male	8
Female	7
Age (years)	27.0 ± 2.7
Anthropometrics	
BMI (kg/m^2^)	21.6 ± 2.1
Fat mass (%)	19.5 ± 4.1
Fat mass (kg)	13.2 ± 3.8
Muscle mass (kg)	51.7 ±9.5
Lean body mass (kg)	54.2 ± 10.1
Laboratory tests	
Haemoglobin (mmol/L)	9.0 ± 0.8
Thrombocytes (×10^9^/L)	244.0 ± 62.9
Albumin (g/L)	42.7 ± 4.2
Creatinine (µmol/L)	80.4 ± 19.4
ALT (U/L)	24.8 ± 9.1
AST (U/L)	25.0 ± 13.4
HbA1c (mmol/mol)	33.5 ± 2.4
Fib-4*	0.67 ± 0.3

ALT, alanine aminotransferase; AST, aspartate aminotransferase; BMI, body mass index; HbA1c, hemoglobin A1c.

* Fibrosis-4 (FIB-4) index for liver fibrosis was calculated based on age (years), ASAT (unit/L), platelet count (10^9^/L) and ALAT (unit/L): (age × ASAT)/(platelets × √ALAT).

### Amino acid exposure and octreotide effects

As previously described (12), baseline glucagon, insulin, glucose, and total amino acids were comparable between visits. At minute 0, glucagon and insulin were lower on the octreotide days than on the Vamin/saline day, consistent with the expected inhibitory effect of octreotide. Total amino acids rose to similar peak levels after Vamin infusion on the Vamin/saline and Vamin/octreotide days, indicating comparable amino acid exposure, while remaining low on the octreotide/saline day. Vamin induced marked increases in glucagon, insulin, and C-peptide, with smaller responses when combined with octreotide and almost no response on the octreotide/saline day. Glucose increased most on the Vamin/octreotide day, rose modestly on the octreotide/saline day, and remained largely stable on the Vamin/saline day. These profiles confirm the successful delivery of the interventions and the intended physiological contrast between study days.

### ACTH and cortisol responses following amino acid infusion are only partly suppressed by somatostatin

Cortisol trajectories were similar across visit days before the amino acid intervention, and baseline concentrations at 0 min were similar between visits (Vamin: 233 ± 26 nmol/L, octreotide: 231 ± 28 nmol/L, Vamin + octreotide: 250 ± 24 nmol/L; mixed-effects model *P* = 0.69) (Fig. 2A). After Vamin infusion, cortisol diverged from the octreotide days, with higher values from ∼30 to 90 min and a clearly greater early response. Peak cortisol was 160 ± 29 nmol/L higher with Vamin than with octreotide (*P* = 0.0003) and 148 ± 23 nmol/L higher than with Vamin plus octreotide (*P* < 0.0001), whereas peaks did not differ between the two octreotide days (−15 ± 18 nmol/L, *P* = 0.68) (Table 2, Fig. 2C). Early baseline-subtracted exposure (bsAUC_0–45_) was likewise greater with Vamin than with octreotide (*P* = 0.0003) and Vamin plus octreotide (*P* < 0.0001), with no difference between the two octreotide conditions (*P* = 0.90) (Table 2, Fig. 2B).

**Figure 2:**
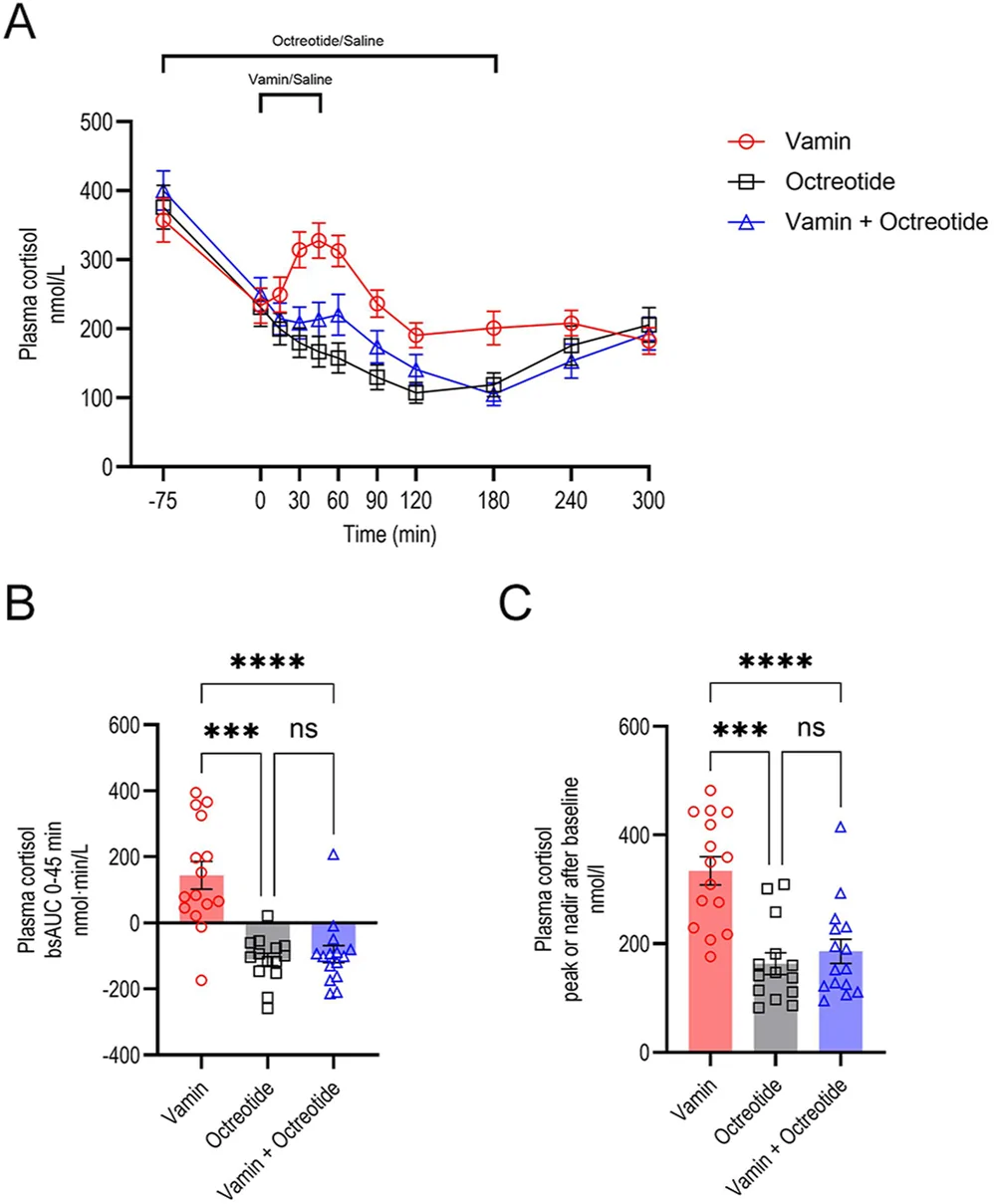
Plasma cortisol response to Vamin and octreotide infusions. (A) Cortisol time–concentration curves. Vamin infusions began at 0 min and ended at 45 min; meanwhile, octreotide infusions began at −75 min and ended at 165 min. (B) Individual bsAUC_0–45_ values. (C) Individual maximal cortisol values, if positive from baseline, or nadir values, if negative from baseline. bsAUCs were computed after baseline correction at time 0 min using the linear trapezoidal method. Brackets show pairwise comparison after Tukey’s correction. Data are presented as mean ± SEM. *P* values <0.05 are considered statistically significant. ***P ≤ 0.001, ****P ≤ 0.0001.

**Table 2:** Hormonal responses during Vamin and octreotide visits. Values are mean differences ± SEM in peak or nadir concentration and baseline-subtracted area under the curve (bsAUC), calculated after baseline correction at 0 min using the linear trapezoidal rule. The exploratory bsAUC windows were bsAUC_₀–₄₅_ for cortisol, bsAUC₀_–_₁₂₀ for ACTH and bsAUC_₀–₃₀₀_ for GH. Between-day *P* values are from repeated-measures mixed-effects models. Outliers identified by the ROUT method (*Q* = 0.1%) were removed.

	Peak or Nadir		bsAUC*	
	Value	*P*-value	Value	*P*-value
Cortisol, nmol/L				
Vamin vs octreotide	160 ± 29	0.0003	239 ± 43	0.0003
Vamin vs vamin + octreotide	148 ± 23	<0.0001	238 ± 40	<0.0001
Octreotide vs vamin + octreotide	−15 ± 18	0.68	−13.4 ± 31	0.9
ACTH, pmol/L				
Vamin vs octreotide	3.5 ± 0.46	<0.0001	4 ± 0.6	<0.0001
Vamin vs vamin + octreotide	1.6 ± 0.47	0.0096	2.9 ± 0.55	0.0003
Octreotide vs vamin + octreotide	1.75 ± 0.39	0.0021	−1.2 ± 0.4	0.027
GH, µg/L				
Vamin vs octreotide	3.5 ± 0.86	0.0045	5.9 ± 1.2	0.001
Vamin vs vamin + octreotide	4.4 ± 1	0.0014	5.6 ± 1.2	0.002
Octreotide vs vamin + octreotide	0 ± 0.01	>0.9999	0.006 ± 0.04	0.99

* Unit for cortisol: nmol·min/L, ACTH: pmol·min/L and GH: µg·min/L.

ACTH concentrations declined before the interventions and were similar at −75 min. At 0 min, baseline ACTH tended to be higher on the Vamin day (3.09 ± 0.36 pmol/L) than on the octreotide (2.10 ± 0.25 pmol/L) and Vamin + octreotide days (2.25 ± 0.41 pmol/L), but this difference did not reach statistical significance (mixed-effects *P* = 0.056). Following Vamin infusion, ACTH increased, whereas it remained low on the octreotide day and rose only modestly when Vamin was combined with octreotide (Fig. 3A). Peak ACTH was 3.5 ± 0.46 pmol/L higher with Vamin than with octreotide (*P* < 0.0001) and 1.6 ± 0.47 pmol/L higher than with Vamin + octreotide (*P* = 0.0096), while peaks were also higher with Vamin + octreotide than with octreotide alone (1.75 ± 0.39 pmol/L, *P* = 0.0021) (Table 2, Fig. 3C). Early ACTH exposure (bsAUC_0–120_) showed the same pattern, with Vamin exceededing both octreotide and Vamin + octreotide (both *P* ≤ 0.0003), whereas bsAUC_0–120_ was slightly lower with octreotide than with Vamin + octreotide (*P* = 0.027) (Table 2, Fig. 3B).

**Figure 3:**
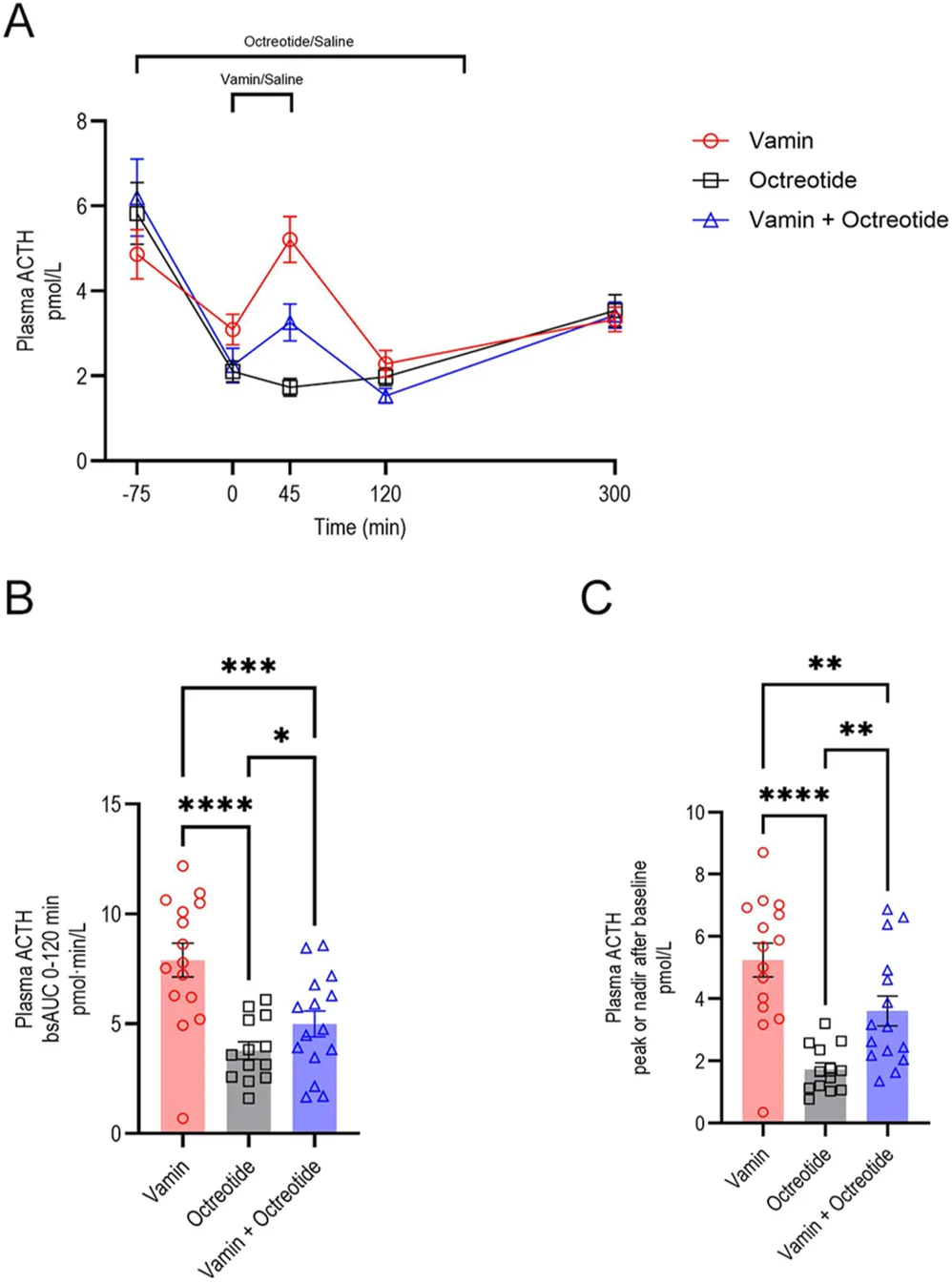
Plasma ACTH response to Vamin and octreotide infusions. (A) ACTH time–concentration curves. Vamin infusions began at 0 min and ended at 45 min; meanwhile, octreotide infusions began at −75 min and ended at 165 min. (B) Individual bsAUC_0–120_ values. (C) Individual maximal ACTH values, if positive from baseline, or nadir values, if negative from baseline. bsAUCs, data presentation, and statistical comparisons are as described in Fig. 2. *P ≤ 0.05, **P ≤ 0.01, ***P ≤ 0.001, ****P ≤ 0.0001.

### The GH response following amino acid infusion is almost completely blocked by somatostatin

GH concentrations gradually declined from -75 to 0 min during the saline and octreotide infusions. At 0 min, after 75 min of saline on the Vamin day and octreotide on the two octreotide days, GH was slightly higher with Vamin (0.90 ± 0.27 μg/L) than with octreotide (0.28 ± 0.09 μg/L) and Vamin + octreotide (0.24 ± 0.07 μg/L). The overall treatment effect at baseline was statistically significant (mixed-effects *P* = 0.046), but no pairwise comparison reached significance after Tukey’s correction (*P* ≥ 0.12). Following Vamin infusion, GH rose sharply, peaking around 120 min, whereas GH remained almost completely suppressed on both octreotide days (Fig. 4A). Peak GH was 3.5 ± 0.86 μg/L higher with Vamin than with octreotide (*P* = 0.0045) and 4.4 ± 1.0 μg/L higher than with Vamin + octreotide (*P* = 0.0014), with no difference between the two octreotide conditions (0.0 ± 0.01 μg/L, *P* > 0.9999) (Table 2, Fig. 4C). Similarly, overall GH exposure (bsAUC_0__–__300_) was greater with Vamin than with octreotide (*P* = 0.001) and Vamin + octreotide (*P* = 0.002), while bsAUC did not differ between the two octreotide days (*P* = 0.99) (Table 2, Fig. 4B).

**Figure 4:**
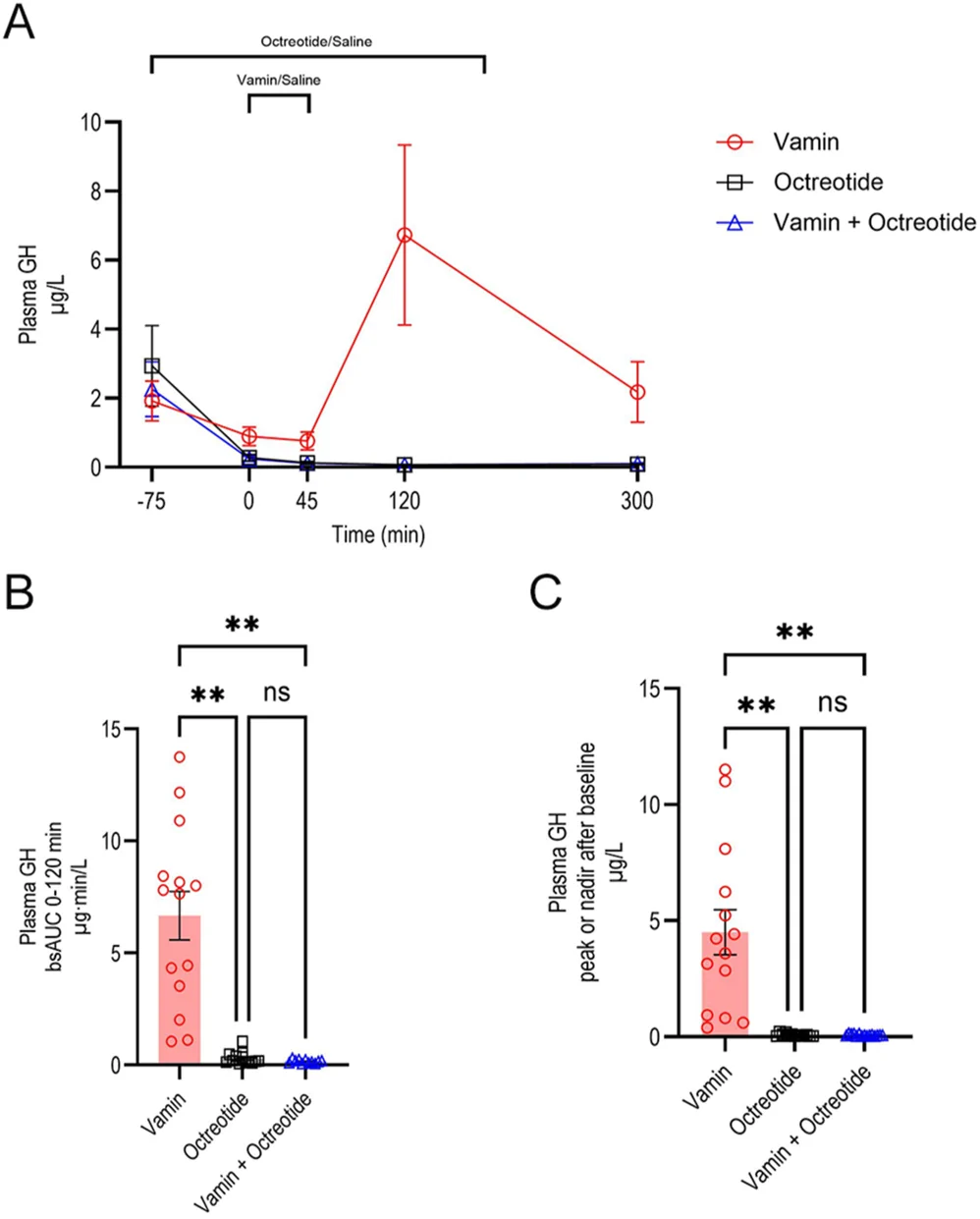
Plasma GH response to Vamin and octreotide infusions. (A) GH time–concentration curves. Vamin infusions began at 0 min and ended at 45 min; meanwhile, octreotide infusions began at −75 min and ended at 165 min. (B) Individual bsAUC_0–300_ values. (C) Individual maximal GH values, if positive from baseline, or nadir values, if negative from baseline. bsAUCs, data presentation, and statistical comparisons are as described in Fig. 2. **P ≤ 0.01.

## Discussion

Our study demonstrates distinct corticotropic and somatotropic response patterns following amino acid infusion, with markedly different sensitivity to octreotide. While somatostatin analogue administration almost completely abolished the growth hormone response to amino acid infusion, ACTH and cortisol responses persisted despite substantial suppression. These findings indicate that activation of the somatotropic axis following amino acid infusion is highly dependent on somatostatin-sensitive pathways, whereas activation of the HPA axis can partly escape somatostatin inhibition.

From −75 to 0 min, ACTH and GH declined more steeply during octreotide than during saline administration. This pattern is compatible with an inhibitory effect of octreotide in addition to the normal morning decline, although the relative contributions cannot be determined from the present design. For cortisol, the already marked diurnal fall of roughly 30 nmol/L per hour in the early morning (13) likely dominates over any additional octreotide effect, which may explain why no clear between-day difference is seen in this interval.

Following amino acid infusion, ACTH and cortisol showed a rapid early response with peaks around 45 min, shortly after the infusion period ended, whereas GH increased more gradually and peaked at approximately 120 min. Given the short circulating half-life of GH (14), this delayed response likely reflects sustained upstream neuroendocrine signalling rather than persistence of the amino acid stimulus itself. The temporal dissociation between the rapid ACTH–cortisol response and the delayed GH peak suggests that amino acids do not act as a nonspecific pituitary secretagogue, but instead recruit partly distinct regulatory pathways controlling the HPA and somatotropic axes. ACTH and cortisol likely reflect a rapid CRH/AVP-mediated response to the amino acid load, whereas the slower GH response may depend on more prolonged GHRH drive or downstream metabolic signalling after plasma amino acid concentrations have begun to decline.

Interindividual variability was substantial, particularly during Vamin infusion, and not all participants showed an increase in ACTH, cortisol, or GH. Variable ACTH and cortisol responses to arginine have also been reported previously, including decreases in healthy individuals (8). This heterogeneity may reflect differences in neuroendocrine responsiveness, together with the pulsatile and diurnal secretion of these hormones. Because responses were calculated relative to the concentration at time 0, participants without a clear stimulus-induced increase could show negative values if hormone concentrations continued to decline during the morning (13).

The near-complete suppression of GH secretion by octreotide contrasts sharply with the persistent ACTH and cortisol responses observed during combined amino acid and somatostatin analogue infusion. This differential suppression pattern suggests that GH secretion during amino acid infusion depends predominantly on somatostatin-sensitive GHRH pathways, whereas activation of the HPA axis can recruit additional mechanisms that are less susceptible to somatostatin receptor-mediated inhibition. Residual ACTH and cortisol secretion during octreotide administration may reflect contributions from AVP-associated signalling, autonomic input, or other upstream neuroendocrine pathways that are not fully suppressed by somatostatin analogue exposure.

A previous report from the same trial found that amino acid infusion partially overcame octreotide-mediated suppression of glucagon, insulin, and C-peptide secretion (12). Although the outcomes do not permit direct quantitative comparison across endocrine systems, the combined findings suggest tissue-specific differences in the balance between amino acid stimulation and somatostatin receptor-mediated inhibition. Pancreatic islet hormone secretion remained substantial during co-infusion, ACTH showed a smaller residual response, and GH secretion was almost completely suppressed. These responses may also be bidirectionally related. Amino acid-induced changes in glucagon, insulin, and glucose may have influenced pituitary hormone secretion, while cortisol and GH may in turn affect glucose metabolism and islet hormone responses. However, because amino acids and octreotide simultaneously altered several endocrine systems, the present study cannot distinguish direct amino acid effects from indirect endocrine crosstalk or determine causal directionality.

Several limitations should be acknowledged. This study was conducted in healthy adults, and the findings may not generalize to patients with an endocrine or metabolic disease. The study did not include a saline + saline visit. Although the pre-intervention period on the Vamin + saline day provided a saline reference from −75 to 0 min, the physiological ACTH and cortisol profiles could not be assessed independently of the interventions after time 0. As this was a *post hoc* analysis of a trial powered for pancreatic islet hormone outcomes, no separate sample size calculation was performed for ACTH, cortisol, or growth hormone, which may have limited the ability to detect smaller differences between conditions. Shift-work status was not systematically assessed and may have contributed to variability in HPA axis measurements. We did not measure upstream hypothalamic mediators, such as CRH, AVP, or GHRH, and mechanistic interpretations therefore remain inferential. In addition, the amino acid mixture contained multiple amino acids, precluding the assessment of the contribution of individual amino acids to the observed endocrine responses.

Our results align with previous work showing that arginine and amino acid mixtures can stimulate GH secretion (5, 6), while their effects on ACTH and cortisol vary across populations and experimental settings (8, 9, 11). Excitatory amino acid signalling has also been implicated in ACTH regulation (10), and somatostatin is a potent inhibitor of GHRH-induced GH secretion, while its effects on ACTH and cortisol are more variable (4). The present study extends this literature by assessing ACTH, cortisol, and GH in parallel within the same octreotide-containing crossover protocol.

Delayed endocrine responses to amino acid exposure may also involve secondary metabolic signalling pathways. Hepatic nutrient-sensing mediators, such as FGF21, have been implicated in endocrine adaptation to altered protein availability (15, 16, 17), although these pathways were not assessed in the present study.

## Conclusion

In summary, intravenous amino acid infusion was followed by rapid activation of the HPA axis and delayed activation of the somatotropic axis in healthy adults. During octreotide administration, the GH response following amino acid infusion was almost completely suppressed, whereas ACTH and cortisol responses were partly preserved, indicating differential somatostatin sensitivity between the two neuroendocrine axes. These findings support the concept that nutrient-derived endocrine stimulation is regulated differently across pituitary systems and provide a physiological framework for studying interactions between amino acid signalling and hypothalamic–pituitary regulation.

## Declaration of interest

NJWA received funding from and served on scientific advisory panels and/or speakers’ bureaus for Boehringer Ingelheim, MSD/Merck, Roche, Novo Nordisk, and Mercodia. The remaining authors have no conflicts of interest to declare.

## Funding

This study was supported by grants from the foundation for the advancement of medical science (Fonden til Lægevidenskabens Fremme, A.P. Møller Foundation) and Marie and Børge Kroghs Foundation. NJWA was supported by the European Foundation for the Study of Diabetes Future Leader Award (NNF21SA0072746), Independent Research Fund Denmark (1052-00003B; 10.46540/4308-00056B; 10.46540/4285-00131B), and Novo Nordisk Foundation (NNF23OC0084970, NNF19OC0055001, NNF24OC0088402, and NNF25OC0105136). The funders of the study had no role in study design, data collection, data analysis, data interpretation, or writing of the report.

## Author contribution statement

NJWA and MMR conceived and designed the study. JR and MMR conducted the clinical experiments. EMSJ conducted the statistical analyses and completed the final data analysis and interpretation. EMSJ drafted the manuscript. NJWA provided intellectual input and was involved in critical discussion and manuscript revisions. All authors reviewed and approved the final manuscript.

## Data availability

Data will be made available upon reasonable request.
